# Venture funding for science-based African health innovation

**DOI:** 10.1186/1472-698X-10-S1-S12

**Published:** 2010-12-13

**Authors:** Hassan Masum, Justin Chakma, Ken Simiyu, Wesley Ronoh, Abdallah S Daar, Peter A Singer

**Affiliations:** 1 McLaughlin-Rotman Centre for Global Health, University Health Network and University of Toronto, 101 College Street Suite 406, Toronto ON, M5G 1L7, Canada

## Abstract

**Background:**

While venture funding has been applied to biotechnology and health in high-income countries, it is still nascent in these fields in developing countries, and particularly in Africa. Yet the need for implementing innovative solutions to health challenges is greatest in Africa, with its enormous burden of communicable disease. Issues such as risk, investment opportunities, return on investment requirements, and quantifying health impact are critical in assessing venture capital’s potential for supporting health innovation. This paper uses lessons learned from five venture capital firms from Kenya, South Africa, China, India, and the US to suggest design principles for African health venture funds.

**Discussion:**

The case study method was used to explore relevant funds, and lessons for the African context. The health venture funds in this study included publicly-owned organizations, corporations, social enterprises, and subsidiaries of foreign venture firms. The size and type of investments varied widely. The primary investor in four funds was the International Finance Corporation. Three of the funds aimed primarily for financial returns, one aimed primarily for social and health returns, and one had mixed aims. Lessons learned include the importance of measuring and supporting both social and financial returns; the need to engage both upstream capital such as government risk-funding and downstream capital from the private sector; and the existence of many challenges including difficulty of raising capital, low human resource capacity, regulatory barriers, and risky business environments. Based on these lessons, design principles for appropriate venture funding are suggested.

**Summary:**

Based on the cases studied and relevant experiences elsewhere, there is a case for venture funding as one support mechanism for science-based African health innovation, with opportunities for risk-tolerant investors to make financial as well as social returns. Such funds should be structured to overcome the challenges identified, be sustainable in the long run, attract for-profit private sector funds, and have measurable and significant health impact. If this is done, the proposed venture approach may have complementary benefits to existing initiatives and encourage local scientific and economic development while tapping new sources of funding.

## Background

Financing the development and deployment of innovative health products to tackle global health problems – including drugs, vaccines, medical devices and new processes – is an ongoing enterprise with diverse participants. For instance, more than 1 billion dollars have been invested by philanthropists and donors into global Product Development Partnerships (PDP’s), and these have shown significant progress in the development of innovative health solutions [1]. Direct financing of global health innovations is being carried out by many others, including many of the G20 nations; United Nations agencies; the Global Fund to Fight AIDS, Tuberculosis and Malaria; philanthropists like the Bill & Melinda Gates Foundation; and multilateral donors and funding initiatives. According to the G-FINDER 2009 report, global neglected disease R&D funding in 2008 came mainly from public and philanthropic donors (87.6%; $2.59B), with a significant minority from the pharmaceutical industry (12.4%; $0.37B) [2].

Yet there remains a health innovation financing gap in Africa, as none of these funds and initiatives have focused on creating sustainably-financed health solutions from domestic science-based health innovation [3]. Funding health innovation in Africa has largely been left to African governments themselves, supported by donors, but this has been done indirectly through small innovation funds like those in Uganda (see the Uganda paper in this BMC series) and similar small funds in Kenya and Ghana. Since these are not specific to health, the bulk of funding has gone to fields other than health R&D which are perceived as less risky. Some international funds also flow into African universities with strong health research programs, such as Makerere University in Uganda and the University of Cape Town in South Africa. While relatively small and not well documented, such funds can play a role in strengthening African health research capacity [4].

One area that has barely been exploited in financing health innovation in Africa is venture capital. Venture capital (VC) refers to provision of finance, managerial oversight and strategic expertise to enterprises with novel, commercially-viable ideas. VC has catalyzed new business models and technologies to deliver novel, high-risk innovations in health in the developed world, and has been an important source of funding for health and biotechnology companies [5]. In the West, venture capital was used to pioneer health products in such companies as Genentech, Amgen, and Genzyme.

Analysis based on empirical data has long raised questions about whether and under what conditions VC firms add sufficient value to justify the ownership stakes they take in fledgling companies [6]. However, they have indisputably been key catalysts in the success of many companies that are now household names, and they bring a range of strengths to developing new science-based businesses. Venture investors can help to identify promising early-stage opportunities, invest in opportunities too risky for banks, support development of new firms, and network new firms with markets and mentors [7]. They develop specialized expertise to select, monitor, and support investments, and thus reduce risk for investors into VC funds. Venture investors may thus be able to transform promising R&D into viable products and services – supplementing public funds by channeling private capital toward global health innovation.

What is the potential of venture funding for supporting science-based African health innovation? The main objective of traditional venture capitalists is to achieve high financial returns, to counterbalance the high risk involved. A complementary objective of other health innovation funders is to achieve high social returns, e.g. improved access to and quality of health products, economic development, and ultimately improved health outcomes. Can these two objectives both be met in designing venture funding for science based health innovation in Africa – whether in a design for health-oriented VC firms, or in a design for an overall health innovation funding system with firms and funders collaborating?

Africa has increasingly been recognized as containing a number of countries offering the potential for high investment returns [8]. In this paper, when we speak of “Africa”, we focus on the more economically and politically stable subset of sub-Saharan African countries which are achieving steady economic growth, deepening democracy, improving governance, and decreasing poverty [9]. We include South Africa, while recognizing that as the leader in the continent in technological and economic terms it has more opportunities and resources for venture funding.

More than 155 public and private venture capital firms managing 273 funds and investments in a wide variety of sectors in 49 African countries were identified in a survey in 2005 [10]; venture activity has grown significantly since then. These funds are a mix of international and domestic venture funds from governments, donors, and the private sector.

However, the majority of existing funds invest in traditional sectors such as agriculture, mining and energy. Except for Bioventures in South Africa, which has invested roughly 12 million US dollars in life sciences, none of these funds has focused on life sciences research and development (R&D) – a sector with long timescales and potentially high technology and market risk [11].

Two issues of concern to investors are risks and the existence of viable opportunities [12]. Yet contrary to international press images of Africa as a land of poverty, war and disease, risks are arguably not too dissimilar to risks experienced by venture capital investors elsewhere in the world [13]. And while poverty and lack of infrastructure and experienced human resources amplify these risks, the growth of African venture capital in other sectors suggests that risks can be addressed through evaluation, monitoring, and risk mitigation steps. (We discuss risks at greater length in the “Observations and analysis” section later.)

Regarding viable opportunities, a 2007 report by the IFC identified health innovation as an investment opportunity in health in Africa [3]. A survey we conducted identified 25 prospective technologies in sub-Saharan Africa outside of South Africa which might be suitable for licensing and further development (see the table in the Introductory article in this BMC series, and [14] for further discussion). An example is Nibima, a product from a traditional plant *Cryptolepis sanguinolenta,* being developed to treat malaria by scientists at the Centre for Scientific Research into Plant Medicine in Mampong, Ghana. Another is a visually-readable portable malaria dipstick test being developed at the University of Ghana, which uses monoclonal antibodies, does not require serum, and is non-invasive. While these examples may or may not prove commercially viable, they do suggest the existence of viable opportunities for venture funds to consider.

In this paper, we offer one of the first studies to systematically examine venture financing as a potential form of funding for African health R&D. We base our analysis on five original case studies of VC firms that have funds invested in health R & D. To our knowledge, this sample contains all health R&D focused venture capital firms based in the developing world created prior to 2007. Three of the firms have operations in Africa: BioVentures (South Africa), Bridgeworks Africa (Kenya), and Acumen Fund (multiple locations in Africa, Asia, and Latin America, with a head office in New York). We complemented these with two additional case studies, one each from China and India: BioVeda Capital (China), and APIDC-VE (India). (Where the firm has only one fund of relevance to this paper, we use the name of the firm to indicate its fund for ease of exposition.)

Funds that focus on health delivery alone were excluded, as these have received attention elsewhere [3]. All case studies were based on a combination of semi-structured interviews with key informants, site visits, and primary and secondary literature analyses. We conducted dozens of interviews with informed consent of fund managers and investees across the five funds and complementary stagnant technology sites, and conducted site visits to the fund and stagnant technology sites between 2007 and 2009; secondary analysis was also done of peer-reviewed literature, articles, news items and web sites. Representatives of the funds were asked to fact-check the case studies; the analysis and interpretation is our own. All quotes are from the interviews unless otherwise noted, and with permission. This study was approved by the Office of Research Ethics of the University of Toronto.

This paper will be of interest to decision-makers in health and development who are open to new approaches tapping private capital, and to entrepreneurs and capital providers who may wish to refine, advocate for, or finance appropriate venture funding. In studying the five venture capital firms, lessons are drawn with respect to considerations for designing African venture funds to support science-based health innovation. Options to support such venture funds are then suggested, including complementary developments such as innovation and convergence centers. The venture funds discussed in this paper, along with the options suggested for creating and supporting future funds, suggest a viable role for VC as a funding source for African health innovation.

## Discussion

### Description of venture funds

In this section, we describe five original case studies of VC firms that have funds invested in health R&D. Three of the firms have operations in Africa; complemented with two additional firms from China and India. These VC firms are subsequently analyzed to draw lessons for future African funds investing in health R&D. The firms are summarized in Table 1.

**Table 1:** 

*Firm Name*	BioVeda China	Bridgeworks Africa	APIDC-VE	BioVentures	Acumen Fund
*Location*	Shanghai, China	Nairobi, Kenya	Hyderabad, India	Cape Town, South Africa	Hyderabad, Nairobi and Karachi, Pakistan
*Total Fund Size*	~$150M	~$2M	~$40M	~$12M	~$50M
*Number of Investments*	12	15	15	8	50
*Key Investors*	IFC, Gov. of Shanghai, Temasek Holdings, HBM Ventures, Lilly Asian Ventures	BioVision Foundation	IFC, APIDC, VenturEast India	IFC, Gensec Bank	Rockefeller Foundation, Cisco Systems Foundation
*Date Founded*	2005	2004	2003	2002	2001

#### BioVeda Capital

Since the early 1990s, international VC funds have existed in China for IT and other sectors. But the BioVeda China Fund, formed in 2005, was the first international venture fund directed at the life sciences in China. This Shanghai-based fund was initially founded by Harvard-trained “sea turtle” (emigrant who returned to China) Dr. Zhi Yang who had over twenty years of biotech and private equity experience, and seven life sciences start-ups under his belt. Initial investors in the original $US32 million Bioveda China fund included the IFC, Temasek Holdings (Singapore) and HBM BioVentures (Switzerland).

BioVeda China has focused on late-stage development and scaling up of existing companies which already have revenues, and as such has characteristics of private equity. However, there are a few investee companies with products in Phase I/II trials. To mitigate risk, BioVeda China Fund’s investee firms span the life sciences – industrial biotechnology, biologics, logistics and medical devices – and share the characteristic of leveraging China’s large domestic demand.

One major challenge the fund has faced is the Chinese regulatory environment.  As founder Zhi Yang noted in 2007, “Knowing people is very important. We keep track and grade all the CEOs [in China].”

BioVeda China brought banks in the United States as co-investors, created a scientific advisory board for scientific and ethical issues, and engaged the local Shanghai government to provide both financial and political capital. A dual-fund model via incorporating in the Cayman Islands allows BioVeda China to navigate Chinese foreign direct investment regulations, and exit its investments internationally. Of BioVeda’s portfolio of twelve investee companies, six have successfully exited and realized returns (4 IPOs and 2 acquisitions).

Although the fund is for-profit, BioVeda China serves as a pioneering model of how international VCs and larger institutional investors can invest in a gateway to R&D in emerging markets. The initial smaller $US32 million fund provided proof of concept for the subsequent funds. In 2007, a second $US100 million fund (BioVeda China II) was raised.

By focusing on late-stage firms with existing revenue streams, diversifying its portfolio, and attracting talented “sea turtles”, Bioveda China demonstrates how investors can pave the way for larger, more high-risk, innovation-focused funds – and shows how a for-profit fund in an emerging economy with a strong research base can create new health technologies.

#### APIDC-VE

The Andhra Pradesh Industrial Development Corporation - VentureEast Biotechnology Venture Fund (abbreviated to “APIDC-VE”) was founded in 2003 in Hyderabad. APIDC-VE is India’s first VC fund focused on biotech. APIDC had previously partnered with the venture group VentureEast for a multi-sectoral VC fund. Having enjoyed prior success with their multi-sector VC fund, APIDC-VentureEast was able to leverage their reputation to raise $US40 million for a biotech-focused fund, through domestic and foreign investors.

The major domestic investors were led by the Andhra Pradesh state government, and the Indian Federal Government’s Technology Development Board which gave $US7.5 million to the project. The key foreign investor was the IFC, which provided technical advice, international networks, and a positive reputational signal that attracted follow-on investors such as CITCO, a global financial service company, and Norfund, Norway’s developmental bank.

The fund was fully invested in 2005, with a planned life of 10 years. It aimed to commercialize Indian biotechnology investments in a for-profit way, but also to have a ripple effect of broader domestic benefits. The fund had some freedom to pursue social benefits through its choice of investees; its public and developmental investors influenced its character.

The fund proactively recruited investments by going into universities years before projects were ready for commercialization. APIDC-VE’s portfolio consists of mostly early-stage companies, but also includes investments outside of India that have relevance to the Indian market. Investees are a mix of R&D and healthcare infrastructure firms.

One innovative investee was Neurosynaptic. The firm specializes in developing telemedicine diagnostic equipment for rural areas, and with a rapidly-expanding network of over 150 centers has the potential to grow into one of the largest such networks in the world. The World Economic Forum selected Neurosynaptic as one of their Technology Pioneers of 2008.

Created in collaboration with IIT Madras, “ReMeDi,” (Remote Medical Diagnostics) is a portable device combining diagnostics for temperature, blood pressure, pulse oximetry and ECG, with telemedical software that transmits information to a doctor for diagnosis. APIDC-VE helped Neurosynaptic partner with the Apollo chain of hospitals to develop a business model targeted at rural communities. Poor villagers cannot afford the time and money to travel for correct diagnosis to large urban centers. Neurosynaptic worked with local organizations to create sustainable franchises that would allow the rural operator to earn the revenues necessary to operate a telemedicine center in their village. 75% of patients who are diagnosed in the village by ReMeDi now get treatment in their village.

The CEO of Neurosynaptic, Sameer Sawarkar, credits APIDC-VE as being distinct from other investors in that they are “looking at this whole segment as a long-term segment that is completely untapped. And they’re willing to experiment and learn new processes that can be applied to the sector as a whole, as opposed to being interested in only investment in the sense of the traditional venture capitalists.” The VCs at APIDC-VE echo these sentiments: “We’ll do a deal in a sector even if we have to create it from scratch… India itself is such a huge market. If we can create products and services for it, we can sustain many funds like [this].”

APIDC-VE illustrates how emerging economies with a strong technical base such as India provide fertile ground for funds. However, funds still need to adapt to local contexts, and look for competitive advantages such as providing a brokering service for large foreign investors. A flexible approach is key when transplanting venture funding. While the fund’s financial results will not become clear until it starts exiting its investments leading up to its 2015 planned ending date, investees like Neurosynaptic suggest promise.

#### BioVentures

CEO Heather Sherwin headed Bioventures, the first life sciences VC in sub-Saharan Africa. A cell biologist trained in South Africa, Sherwin persuaded her financial services employer that they needed a biotech VC fund to complement their successful IT VC fund. This 80 million rand (~$US 12M) biotech fund made initial investments in 8 home-grown companies between 2002 and 2004, with most of the investments having been exited by 2009.

Seed financing came from the IFC. Bioventures was reportedly the IFC’s first-ever biotechnology fund investment. Bioventures’ investees are focused on R&D-intensive medical devices, drugs, and natural products – categories chosen partly due to South Africa’s inability to compete with China and India on manufacturing costs for less R&D-intensive products.

One such investee, Disa Vascular, is now a global player with over 30 employees and several million dollars in annual revenue. The firm produces several niche products for treatment of coronary and peripheral artery disease, including coronary stents and diagnostic catheters that are marketed in the European Union. Notably, Disa conducts clinical trials in South Africa – a testament to its ability to attract clinician interest despite the strong presence of multinationals. Bioventures helped Disa by raising operating capital, advising on a niche R&D-intensive strategy with high market entrance barriers, and linking them to international partners. Follow-on financing of ~$US1 million came from international investors such as Lacuna SICAV (Belgium) in 2007.

Other successful investees included PlatCo and Shimoda, two drug development companies focused on platinum-based anti-cancer compounds and enhanced generics respectively. Both were reportedly sold to US-based Abraxis BioScience in 2008 for $US15 million.

While the fund had a for-profit focus, its strategies and learnings are applicable to future funds with social impact aims. It actively sought out exciting R&D, rather than waiting for business plans to come to them – 8 of 300 potential investments were supported, with investments sought upstream in universities. The fund used risk-mitigating mechanisms including provisions to increase its equity share if an investee didn’t meet previously-agreed performance milestones – a shock for entrepreneurs used to milestone-free government funding. Challenges included competing against multinationals with higher credibility and funding, the relative lack of an entrepreneurial culture in South African research institutes, and exchange controls that made selling companies internationally difficult.

The small fund size prevented follow-on investments into investee companies, which meant Bioventures was forced to give up equity, or to sell investee companies prematurely to international purchasers. Risk-averse local investors combined with long timeframes and high risk associated with the life sciences made local follow-on investments difficult, especially since some investors had unrealistic expectations about financial returns.

Bioventures illustrates how life sciences VC can work in strong sub-Saharan African economies. At the same time, the fund’s experience shows how achieving social impact is more challenging. One investee had a promising HIV/AIDS drug discovery platform in addition to a pain program. Eventually the fund and the firm concluded that they could not afford to keep siphoning money to R&D. As CEO Sherwin said, “We canned the AIDS program in favor of pain and oncology programs whose IP could be sold to international investors…If you follow your pure VC mandate, you cannot invest in TB or HIV companies. You’re not going to get your returns.”

Two key lessons from Bioventures were the fund’s inadequate size, and the fund’s inability to pursue mixed-return investments due to the kinds of investors the fund had. Sherwin suggested “mixed funds” as the way forward – funds that would have investment capital with a lower expectation of financial return, and benefit from technical assistance and other support that would make investing into areas targeting health impacts more feasible. (For further details, see the Bioventures paper in this BMC series [15].)

#### Bridgeworks Africa

Bridgeworks Africa was the first Kenyan life sciences focused VC fund. However, unlike traditional VC models, Bridgeworks opted to take a non-profit approach and partner with a single institution, the International Centre for Insect Physiology and Ecology (ICIPE). As the brainchild of Dr. Hans Herren, ICIPE’s Director from 1994 to 2005, Bridgeworks hoped to commercialize the rich research base that ICIPE had developed since its inception in 1970 through partnerships with researchers from over 70 countries.

ICIPE’s focus was on developing tools and strategies for managing arthropods to benefit the health of Kenyans. Particularly noteworthy were its attractant-repellent technologies for controlling tsetse flies and mosquitoes, which are the biological vectors for agents causing trypanosomiasis and malaria. The Institute reportedly had 8 patents related to these technologies.

Bridgeworks Africa received seed funding of $US1.5 million in 2004 and spun off from the Swiss-based BioVision Foundation that ICIPE’s then-Director Herren had founded in 1998. What began as a promising experiment became mired in problems. As of 2009, Bridgeworks Africa had restructured, and the original operation was no longer active.

Raising capital was challenging due to lack of interest by the government, stringent criteria of international development banks, and lack of linkages to the Kenyan diaspora. It was difficult to reconcile traditional NGO models with Bridgeworks’ new “social VC” model. Bridgeworks faced resistance from the researchers of ICIPE, weak institutional frameworks, and regulatory inefficiencies in moving projects forward.

The fund may have also spread its limited resources too thin. It distributed its scarce capital across fifteen investments, and had some of its senior staff managing investments remotely from Switzerland. By associating itself primarily with one research institute, Bridgeworks may have had limited exposure to the best investment opportunities.

Many of ICIPE’s most promising technologies such as its attractant-repellent technologies simply did not have scalable business models, especially since their target market included nomadic and rural communities. Although Dr. Hassanali, one of the ICIPE’s lead attractant-repellent researchers, believed that involving companies would increase the pace of dissemination and get “faster and widest impact," the profits were not significant.

“ICIPE has focused largely on small-scale farmers. That was the philosophy right from the start… I said ‘Let’s develop all these products targeting different economic strata in Eastern Africa… [Bridgeworks Africa] didn’t show too much interest… In some cases, it’s better to let communities take over everything. In other cases these technologies need entrepreneurs, but they need to be of a special kind – entrepreneurs who don’t want to be millionaires or billionaires overnight.”

Bridgeworks Africa illustrates the extreme challenge of operating a VC fund in sub-Saharan Africa. The fund achieved modest success in evaluating and identifying technologies, but was unable to capitalize on this. Funds ignore at their own peril the risk of overreaching with limited resources, and the importance of adapting business models to local contexts.

#### Acumen Fund

Acumen Fund is a social VC fund which invests for both social and financial returns. It has offices in New York, India, Pakistan, and Kenya, and a significant focus on health. Founded in 2001 by Jacqueline Novogratz, a former investment banker with a passion for microfinance, Acumen initially provided grants to R&D-focused organizations. However, these technologies failed to reach the market, which led Acumen to focus on distribution and delivery, and shift towards market-informed investing and a more hands-on approach.

Acumen believes that well-crafted equity coupled with metrics “forces the financial discipline necessary for social enterprises to achieve sustainability.” Indeed, this strategy has paid off. Its investees now span the entire health system from manufacturing to financing to delivery, and some have become global players.

One investee, Botanical Extract EPZ Limited (BEEPZ, formerly ABE) works with 3,500 farmers in East Africa producing artemisinin for 60 million doses of malaria treatment yearly. The cost of artemisinin combination therapies is more than that of conventional drugs due to lack of supply; BEEPZ is helping to resolve this supply crisis. Another, A to Z Textile Mills, produces 25 million long-lasting bed-nets per year that provide protection to nearly 40 million people, while employing 7000 Tanzanians. (See the A to Z paper in this BMC series.)

The fund recognizes that it may take longer for investments with social impact to provide a return on investment, and may require more support. Its innovative fellow and alumni programs tap talent from the developed world to support investees and develop management, providing inexpensive yet experienced advice. Acumen’s local presence through subsidiaries in India, Kenya and Pakistan allows it to monitor local opportunities and conditions. Its rigorous selection methodology and hands-on approach to training human capital and designing business strategies have helped achieve remarkable social impact, as quantified by metrics such as the tens of millions helped through investees like BEEPZ and A to Z. (For more on Acumen Fund’s health investment portfolio and process, see [16].)

### Observations and analysis

Based on the above case studies and subsequent analysis, we discuss design considerations for potential African venture funding for science-based health innovation. (Our analysis complements other initiatives for African funding for science-based health innovation, e.g. [17].)

#### Types of venture firms

Based on the above cases, three categories of venture capital firms can be identified, based on their approach and goals:

1.*Private VC (pure financial)*. Independent venture capitalists structured as corporations, with a primary aim of financial returns (Bioventures and Bioveda China).

2.* Public VC (hybrid financial and development).* Publicly-financed venture capitalists consisting of supra-national or government funded venture capital programs, managed by professional venture capital managers (APIDC-VE). Along with financial returns, they have a mandate for local economic development.

3.* Social VC (hybrid financial and social).* Social venture capitalists like Acumen Fund have a mandate to invest for combined financial and social returns. Bridgeworks Africa had a similar combined mandate, though it achieved less success.

A common investor for all venture firms except Acumen Fund and Bridgeworks was the International Finance Corporation (IFC), and the IFC has co-invested with Acumen Fund. All firms operated within a single country, except for Acumen Fund which has country offices; understanding local business and regulatory contexts well enough to develop new technology solutions may require a deep level of local knowledge and experience

#### Types of fund risk

When considering whether to set up or invest in a health venture fund in Africa, numerous risks become apparent. These risks can be divided into three categories, relating to sector, location, and firm. (Risks relating to particular investments of a health venture fund are considered briefly in the next section. A broader view of barriers to health technology R&D in the developing world can be found in [18] and [19].)

Sector: investing in R&D anywhere in the world is a risky proposition almost by definition. Many new health technologies pose particular difficulties due to lengthy time scales and regulatory and clinical requirements. Within the health technology sector, neglected disease R&D suffers from a lack of profitable markets.

Location: risks have been perceived as especially high for most of the African continent, though this is changing for the best-governed countries [9]. Challenges of bureaucratic inefficiency, corruption, and poor infrastructure endure. There is little early-stage government funding to stimulate innovation activity and de-risk private and VC investment, though this is gradually changing in some countries such as Uganda. With respect to financial markets, it may be difficult for international investors to repatriate funds; this was an issue identified by Bioventures management, with respect to exchange controls. There has been a lack of IPO and other exit opportunities on the continent. There is also a local lack of experienced life science entrepreneurs and mentors.

Firm: there may be a trust problem if the venture firm is itself new and unknown to investors – it is interesting to note that all venture firms studied had management with substantial experience in developed countries. Fund lifespans may be an issue, as well as small fund sizes which limit capacity. Both Bridgeworks and Bioventures faced challenges due to limited capital for initial and follow-on investments; the firm management felt that their success had been concomitantly limited.

#### Investment strategy

The type of investments that these firms made varied widely, ranging from R&D in biotechnology to investments in pharmaceuticals, medical devices, and manufacturing. Each type of investment has its associated risks and rewards, and these must be considered in detail to assess a fund’s viability. Such work is already underway with respect to particular areas, such as pharmaceutical innovation in Africa [20]; initial work on several other health sectors has also been done [3].

The stage of investment differed between cases. While Bioveda focused on late-stage development of existing companies, Bridgeworks and Bioventures provided early seed capital to their investees, often investing in new companies built around technologies rather than in existing companies.

The venture capital firms, already themselves constrained by lack of resources, had to be more involved in developing investees than might be typical in North America and Europe. This naturally requires more time and expense from fund management, reducing the financial return of the fund, and thus making venture funding for global health more difficult [21]. One avenue for mitigating this in the future may be developing third-party networks, knowledge resources, and technical assistance programs to support investees – these along with national funds can augment services the investor can provide.

A look at the firms’ investments suggests that they had non-trivial health impact, but how much is difficult to quantify. Metrics for health impact are an area for further research. Acumen Fund, as a social investment firm, has done the most to attempt to develop metrics for its social returns, including health impact.

The investment strategy itself was not an unconstrained choice, but rather an evolved compromise driven by fund size, local investment pipeline, and the mandate and expectations of investors. Many opportunities were not available locally, and would not have been in the short term even if additional funds had been available. All this underscores the importance of mapping local strengths and support mechanisms, to generate a data-driven landscape within which realistic venture funding models can be designed.

For future funds, social and financial goals of investors along with a data-driven landscape of the characteristics of investment opportunities will guide investment strategy. This includes return expectations for invested capital; the amounts, stages, and conditions of investment; the third-party support required before investments are made; and market assessments of technologies, including affordability and ability to compete with existing technologies.

#### Investor scale and type

The investor scale and type is a key design consideration, and we consider several natural possibilities below.

Private-sector financing: A pure private VC approach to health R&D is unlikely to work for several reasons: time and expense required to identify and support investees; a relatively sparse pool of potential investees; difficulty in exiting investments; location and R&D risk; and opportunity costs as compared to venture investments in other sectors and locations. However, the private VC model may be relevant for late-stage, smaller investments such as medical devices to facilitate healthcare delivery – possibly linked with existing healthcare funds like the $57M Aureos fund which is investing in healthcare delivery such as clinics, for which there is established demand in numerous African countries [22]. Such delivery-facilitating investments could be similar to that made by APIDC-VE (India) in NeuroSynaptic’s ReMeDi, for remote telemedical diagnosis; they could also be import substitutions to make or modify commonly-used, commodity medical products locally or more cheaply.

Institutional financing: Financing raised from various sources and disbursed for research institutions has supported commercialization in the developed world, such as Karolinska Development linked with the Karolinska Institute (Sweden) – a roughly $250 million (USD) fund with a life sciences focus. In Kenya, KEMRI constructed a manufacturing facility with Japanese funds to produce blood kits for the local market (see the KEMRI paper in this BMC series). In Nigeria, NIPRD was the first in a series of partners developing compounds for sickle cell anemia (see the Niprisan paper in this BMC series). However, even the largest African research institutions appear to lack the size, experience, and local supporting expertise to sustain institution-specific funds capable of making larger investments. We hypothesize that institutional financing may have a small and gradually increasing role at a few select institutions with strong R&D capacity and international partnerships that mitigate risk.

National financing: The US VC industry has been partly catalyzed by US government grants from organizations like the National Institutes of Health. In the last decade the South African government established several funding programs to stimulate technology innovation in the country, most notably the Innovation Fund and the Biotechnology Regional Innovation Centres (BRICs). These centers were charged with investing national funds into technology innovation, providing support services, investing at the high-risk early stage, and de-risking projects for private sector players including VCs. Recently, these programs along with several others were drawn together to form the Technology Innovation Agency, which hopes to better coordinate the national funding of technology innovation in South Africa. In Uganda, new government funds for supporting science-based innovation have been started (see the Uganda paper in this BMC series). We hypothesize that national financing has a role to play in providing early-stage funding, and enabling capital flow – galvanizing private-sector and international venture funding, not least by developing research ideas to a more “investable” stage.

International financing: Virtually all the advanced health-related projects presented in this BMC series had involvement from a development or multilateral agency. International partnerships allow African scientists to raise the quality of their R&D and gain support. Competitions such as the Grand Challenges Explorations initiative of the Bill & Melinda Gates Foundation are open to individuals worldwide; unconventional approaches are supported by rapid response to grant applications, and initial seed rounds of $100,000 with potential follow-on funding of up to $1 million [23]. Other foundations such as the Rockefeller Foundation and Wellcome Trust have made significant investments into African life sciences research initiatives. The Africa Enterprise Challenge Fund is making venture investments into research-based agribusiness, renewable energy and rural financial services, supported by developmental finance organizations. Acumen Fund’s “social venture capital” approach employs “patient capital” to nurture start-ups with long-term support, with success to date in low-cost healthcare delivery and manufacturing enterprises such as BEEPZ (artemisinin) and A to Z Textile Mills (bed nets). We hypothesize that international financing can support science-based R&D where a commercial business case may not be evident, especially if assisted by appropriate metrics, milestones, networks, and platforms.

#### Drawing on a range of actors

Successful venture funding for African science-based health innovation will benefit from a multi-partner approach involving institutions, public and private sector funding, successful African entrepreneurs, and international agencies. A precedent may be the vaccine innovation landscape, which involves: institutions and public-private partnerships for R&D; public sector funding for purchasing; international financing through e.g. GAVI, UNICEF, the Bill & Melinda Gates Foundation, and Advanced Market Commitments; and entrepreneurs for local development, manufacturing, and delivery of key vaccines. All players are necessary to create an incentive landscape where venture financing can invest for high financial and social returns.

More research and engagement with venture funding groups is required to clarify incentives, partnerships, and funding structures that make venture funding for global health attractive, and to distinguish health investments for which venture funding will be appropriate [24]. Establishing examples and eventually a track record of success will be important; the five cases discussed earlier may be modestly suggestive in this regard.

Further investigation is also required into the relative roles of financial and social returns. Even the venture funding groups discussed above had differing ratios of financial to social return goals. These differences will become yet more evident when a range of private-sector, institutional, national, and international actors must interoperate to support science-based health innovation. We suggest developing better metrics for social and health returns, and bringing such metrics into common use by all actors, joining existing financial metrics. In this way, a common language can be used when discussing the motivations of each actor, and the impact of their respective actions.

## Summary

From the case studies and analysis above, we believe that there is an argument for using the venture approach as one component of developing drugs, vaccines, diagnostics and medical devices in Africa. A life sciences Africa VC fund should be structured to mitigate the risks discussed, attract for-profit private sector funds, and provide managerial and financial oversight and training to start-up companies. Attracting successful African entrepreneurs from other sectors into the fund will be valuable, as will tapping experienced venture investors both domestically and abroad.

Based on the types of venture firms and fund risk identified, an investor mix of for profit and not for profit is a likely scenario, supported by domestic and international financing sources – all of which can create an incentive landscape which can both be attractive to and benefit from venture funding.

Considerations of investment strategy and investor scale and type will be critical. For example, an anchor investor might be a not for profit institution that is interested in health impact, but keen to establish a sustainable life sciences commercialization model. This anchor investor would mobilize seed funds that would mitigate the real and perceived risk associated with investing in early stage life sciences projects. A second-tier investor group could be a consortium of larger investors with the shared goal of providing “patient capital” to scale up the fund’s best opportunities. Local investors and investees should also have a stake in the fund’s success.

Funds should seek to involve private-sector partners at later stages, to create self-sustaining flows of capital focused on innovation in Africa. Government policies should be aligned through R&D and investment support, and general good governance. Drawing on a range of actors will be essential.

A model to encourage commercialization of health products in Africa through the development of “Convergence Centers” has been proposed [25], and subsequently discussed in the broader context of S&T development [26]. These Centers, which would co-locate entrepreneurs, researchers and capital providers, would be useful vehicles through which a venture funder could identify technologies. They would act as a one-stop shop for technologies that scientists in African countries are developing, as well as a physical and virtual hub for training and connecting talent.

Based on the cases and analysis in this paper, there is a venture funding niche for science-based African health innovation, with opportunities to make profits as well as a social contribution. Such funds should be structured to overcome the challenges identified, be sustainable in the long run, attract for-profit private sector funds, have measurable and significant health impact, and demonstrate a different approach than aid-based international development. While a range of supportive policies and actors are required to tackle health challenges in Africa, we believe the proposed venture approach can play a significant part in encouraging local scientific and economic development while tapping new sources of funding.

## Funding

Funding for this project is from a grant from the Bill & Melinda Gates Foundation through the Grand Challenges in Global Health Initiative.

## Competing interests

PAS is on the scientific advisory board of BioVeda China 2 Fund, and he was an advisor to the IFC (International Finance Corporation), both for compensation.
